# Young Men’s Association

**Published:** 1874-12

**Authors:** 


YOUNG MEN'S ASSOCIATION.

The young men of St. Peter's Church, West Twentieth Street, have an organization for charitable purposes, which is doing very much good. Its president is Mr. A. M. Lesley, a gentleman who is active in all good works, and who takes a deep interest in the work of the excellent charity of which he is the head.

The Association has been in existence for fifteen years, and has been productive of great good during that prolonged period. Its objects are to go among the poor of the parish district ; to relieve, in such ways as may be within its power, those suffering from sickness or other distress ; to bring the children into the Sunday School ; to induce persons to come themselves to hear the Gospel preached on the Lord's Day ; and to carry out, in some degree, among this numerous class, the great design of the Church. This work is undertaken as a slight assistance to the Rector, and under his direction.

The funds with which the charitable work of the Association is done are derived from donations, and from collections made in the Church on the first Sunday evening in each month.

During the hard winter which is upon us, the demands upon this charity will be very large ; and we feel that we cannot do better than to suggest to our readers, who have either money or articles of use in the household which they can spare, that they leave them with Mr. Lesley, at his store, No. 224 and 226 West Twenty-third Street, who will give his personal attention to their careful distribution.

